# Herbaspirillum seropedicae Bacteremia Secondary to Pneumonia in a Patient With Chronic Obstructive Pulmonary Disease

**DOI:** 10.7759/cureus.59573

**Published:** 2024-05-03

**Authors:** Alex Hu, Xiangliang Sui, Joan Tao, Jeramey Stewart

**Affiliations:** 1 Emergency Medicine, University of Cincinnati College of Medicine, Cincinnati, USA; 2 Emergency Medicine, Philadelphia College of Osteopathic Medicine, Philadelphia, USA; 3 Radiology, University of Missouri School of Medicine, Columbia, USA; 4 Internal Medicine, St. Luke’s University Health Network, Chesterfield, USA

**Keywords:** herbaspirillum seropedicae, opportunistic infection, chronic obstructive pulmonary disease, pneumonia, bacteremia, copd, microbes, infectious disease

## Abstract

*Herbaspirillum seropedicae* is a species of bacteria commonly found in vegetation, but in rare cases, can cause opportunistic infections in human hosts. Infections typically occur due to environmental exposure to the pathogen, such as through agriculture or gardening. However, these incidents typically only involve immunocompromised patients. Our present report describes a case of sepsis secondary to pneumonia in an adult with a history of chronic obstructive pulmonary disease who presented with complaints of shortness of breath and hypoxia. Although initially misidentified as *Burkholderia cepacia*, blood culture and reference lab eventually confirmed *H. seropedicae* bacteremia. The patient was admitted for treatment with intravenous antibiotics with significant improvement and subsequent discharge. *H. seropedicae *is often clinically misidentified due to its rarity. As we observe the increasing pathogenicity of *H. seropedicae*, clinicians must be better prepared to recognize the symptoms of its infection. Technologies such as matrix-assisted laser desorption/ionization time-of-flight mass spectrometry have proven to be useful in distinguishing *H. seropedicae* from other similarly presenting species.

## Introduction

*Herbaspirillum seropedicae* are Gram-negative rods belonging to the Oxalobacteraceae family. They are oxidase-positive, non-fermenting, and nitrogen-fixing bacteria found in soil environments as part of the plant rhizosphere. As a nitrogen biofertilizer, *H. seropedicae* produces phytohormones that help stimulate the growth of other plants, thus playing an important role in growing crops, such as maize, rice, sorghum, and sugarcane [1]. However, there has been growing evidence of *H. seropedicae* transitioning from environmental to pathogenic bacteria. A 2019 genome comparison study revealed that strains isolated from human infections had lost genes related to nitrogen fixation and gained genes for lipopolysaccharide biosynthesis, with the incorporation of sialic acids to evade the human immune system [2]. While still rare, cases of *H. seropedicae* colonizing human hosts as opportunistic infections have been reported, primarily affecting patients with predisposing conditions such as cystic fibrosis or those undergoing cancer treatment with immunosuppressive medications [3-5]. Occasionally, these patients also have a well-documented history of environmental exposure before infection, such as gardening, cleaning ponds, and aquatic exposure [3,6]. We present the case of an elderly patient with multiple respiratory risk factors and a known history of environmental exposure who was diagnosed with sepsis secondary to *H. seropedicae* pneumonia.

## Case presentation

A 74-year-old male with a past medical history of chronic obstructive pulmonary disease (COPD) (emphysema) on inhaled corticosteroids and 2 L nasal cannula oxygen supplementation at baseline, pulmonary fibrosis, thoracic aortic dissection with replacement, atrial fibrillation, coronary artery disease status post-stenting, hypertension, and pericarditis presented to the emergency department due to a chief complaint of shortness of breath. The patient reported experiencing shortness of breath after waking up in the morning when he noticed his oxygen saturation was hypoxic in the 60s. In response, he increased his oxygen supplementation to the maximum of 6 L which improved his oxygen saturation to 70%. Given that he was still hypoxic, the patient presented to the emergency department for evaluation. The patient also reported experiencing peripheral edema and weakness and endorsed the development of a fever of 100°F with some chills during the prior night. He denied experiencing hemoptysis, wheezing, nausea, or vomiting. The patient reported that he lived with his wife and denied any known sick contacts. When asked about daily activities, the patient commented that he was an avid gardener. Upon arrival at the emergency department, the patient exhibited an oxygen saturation of 81%, which improved to 90% after 4 L of oxygen supplementation.

**Figure 1:**
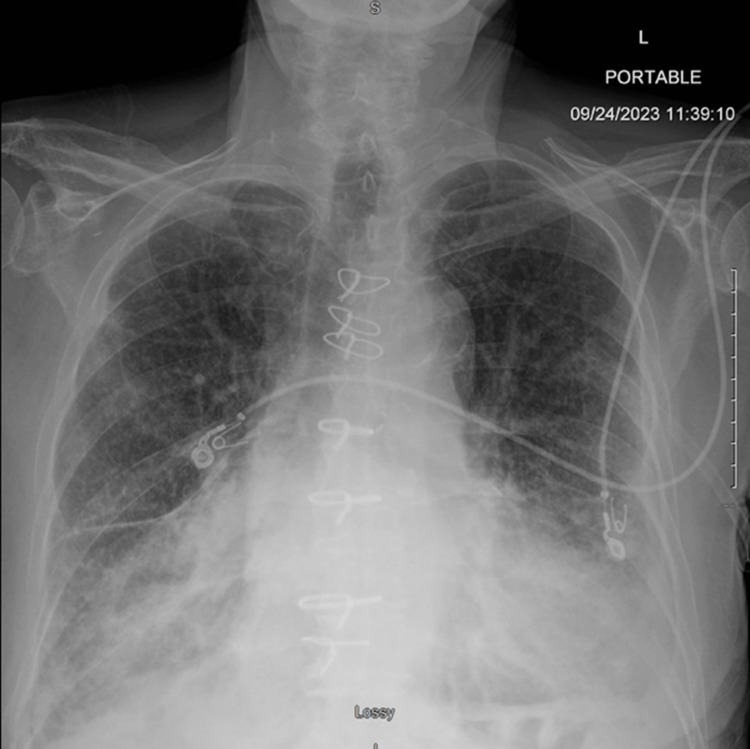
Initial patient chest X-ray. Cardiomegaly with interstitial accentuation which is more prominent in the bases symmetrically. Probable small left greater than right effusions suggestive of congestive failure. This is on a background of probable pulmonary fibrosis.

**Figure 2:**
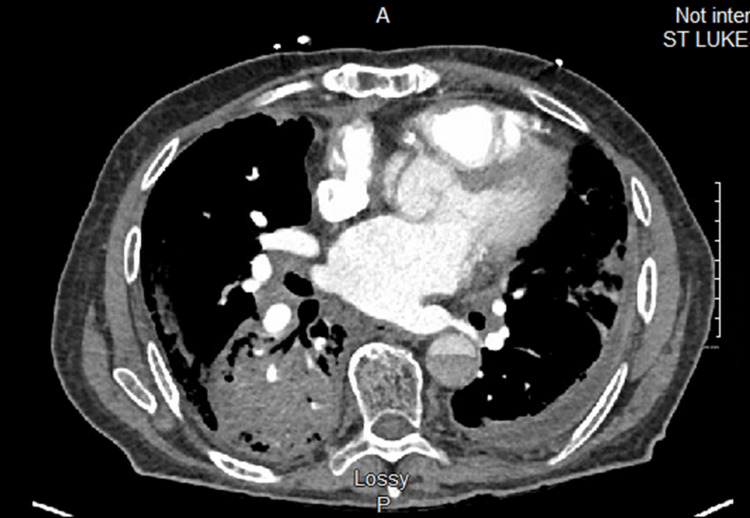
Initial patient CT angiography. Opacification of the pulmonary arteries without filling defect identified to suggest pulmonary emboli. There is a new dense consolidation in the right lower lobe. There is a slight increase in the amount of infiltrate in the right upper lobe posteriorly and the right middle lobe inferiorly. The prior left upper lobe infiltrate has improved. A stable amount of bilateral pleural effusion can be noted. Ascending aortic repair is stable. Arch and descending aortic dissection are again identified, continuing into the upper abdomen, stable in appearance. The visualized portions of the upper abdomen appear unchanged.

A chest X-ray showed cardiomegaly with interstitial accentuation that was symmetrically more prominent in the bilateral bases and probable small left greater than right effusions that were suggestive of congestive failure (Figure 1). CT angiography imaging of the patient’s chest was negative for pulmonary embolism but revealed a new area of dense airspace infiltrate in the right lower, right middle, and right upper lobes, in addition to bilateral pleural effusion (Figure 2). Tracheal aspirate was found to be positive for methicillin-resistant *Staphylococcus aureus*. The culture of the patient’s sputum yielded very light growth of *Pseudomonas aeruginosa*. The patient’s blood culture revealed Gram-negative rods that were confirmed to be *H. seropedicae* by the reference lab.

The patient was ultimately diagnosed with severe sepsis secondary to bacterial pneumonia, acute-on-chronic hypoxic respiratory failure, combined emphysema and pulmonary fibrosis, and pulmonary hypertension. The patient was admitted to the hospital; started on cefepime, azithromycin, and vancomycin; and infectious disease was consulted. Per infectious disease recommendations, the antibiotic regimen was narrowed to cefepime 2 g intravenously (IV) every eight hours. The patient improved significantly throughout his hospital stay and was discharged on day eight with a peripherally inserted central catheter line in place to finish the two-week course of IV cefepime at home. At the time of discharge, the patient required 6 L of supplemental oxygen and was given instructions to use an oxygen concentrator.

## Discussion

*Herbaspirillum* spp. are ubiquitous organisms commonly found in the rhizospheres of crops that have recently transitioned to human hosts through opportunistic infections caused by handling materials inoculated with the species [3]. For instance, our patient endorsed gardening as a hobby, and his handling of plants and soil may have contributed to his condition. Manifestation of infection depends on the type of exposure, and the genus has not shown limitation of infection within a particular body system. While members of the *Herbaspirillum* genus are often implicated with lower respiratory tract infection, they have also been found in infections of the urinary tract and the integument [6,7]. These cases typically involve patients who are vulnerable to microbial infection and rarely affect healthy individuals. *Herbaspirillum* spp. most commonly infects immunocompromised patients or patients who have chronic pulmonary conditions, such as cystic fibrosis, which facilitate microbial growth [3]. However, outcomes have generally been favorable, and the bacteria are readily eliminated through the application of IV antibiotics [3,5,6].

Our patient suffered from emphysema, which is one of the conditions that comprise COPD. Emphysema is characterized as damage to the pulmonary structures distal to the terminal bronchiole, including the cilia and alveoli [8]. This damage is often caused through inhalation of noxious gases, and emphysema is often found in patients who smoke cigarettes. The destruction of these tissues results in the recruitment of inflammatory cells, which release proteinases that lead to mucus hypersecretion [8]. This combination of mucus hypersecretion and impaired clearance capability of the pulmonary cilia creates an environment of mucus stasis that facilitates microbial growth. As such, emphysema as a risk factor likely contributed to the colonization of *H. seropedicae* within our patient’s lungs, subsequently leading to bacteremia. Furthermore, the patient’s COPD medications included daily inhaled corticosteroids in the form of nebulized budesonide and budesonide/formoterol/glycopyrrolate inhaler. As an immunosuppressive medication, inhaled corticosteroids are associated with a 69% increased risk of developing pneumonia according to a large cohort study [9]. Although budesonide is associated with a lower risk of pneumonia compared to other inhaled corticosteroids such as fluticasone, budesonide still mildly increases the risk of pneumonia and may have contributed to the development of *H. seropedicae* pneumonia in our patient.

Before confirmation with matrix-assisted laser desorption/ionization time-of-flight mass spectrometry (MALDI-TOF MS), our patient was suspected to have bacteremia secondary to *Burkholderia cepacia*. Due to phylogenetic and phenotypic resemblance, *Herbaspirillum* spp. is commonly misidentified for *Burkholderia* spp., which may mask the true prevalence of infection by *Herbaspirillum* spp. For example, in a study analyzing bacterial colonies cultured from the sputum of patients with cystic fibrosis, *Herbaspirillum* spp. was misidentified as *B. cepacia* complex in 19 of 28 patients (68%) [5]. Key differences between the genera include antimicrobial susceptibilities, prognosis, and transplantation considerations. *Burkholderia* spp. tends to be multidrug resistant with poorer outcomes, whereas *Herbaspirillum* spp. appears to be readily eradicated and has a favorable prognosis. Perhaps more importantly, many treatment centers consider *Burkholderia* spp. infection an absolute contraindication to lung transplantation due to especially poor outcomes, thus denying patients with end-stage pulmonary disease the sole option for survival [5]. The increased availability of newer molecular methods such as MALDI-TOF MS should allow laboratories to correctly identify *Herbaspirillum* spp. MALDI-TOF analysis demonstrates one peak at m/z 6,701.54 shared by all *Herbaspirillum* strains tested [10].

## Conclusions

As we observe an increasing transition of *Herbaspirillum* from environmental to pathogenic bacteria, characterized by genetic changes favoring pathogenicity, it becomes necessary to broaden our clinical suspicion and consider these atypical pathogens in patients with risk factors such as immunocompromised states or significant environmental exposures. Notably, in this case, the patient’s COPD, environmental exposure through gardening, and the use of immunosuppressive inhaled corticosteroids were identified as predisposing factors for *Herbaspirillum* infection. The misidentification with *B. cepacia* before MALDI-TOF MS highlights the importance of accurate identification methods. Moving forward, the consideration of *Herbaspirillum* spp. in patients with immunocompromised states, history of environmental exposure, or *Burkholderia* diagnoses may require confirmatory MALDI-TOF MS analysis for accurate differentiation and optimal clinical management.
